# Misuse of the Michaelis–Menten rate law for protein interaction networks and its remedy

**DOI:** 10.1371/journal.pcbi.1008258

**Published:** 2020-10-22

**Authors:** Jae Kyoung Kim, John J. Tyson

**Affiliations:** 1 Department of Mathematical Sciences, Korea Advanced Institute of Science and Technology, Daejeon, Republic of Korea; 2 Department of Biological Sciences, Virginia Tech, Blacksburg, Virginia, United States of America; 3 Division of Systems Biology, Virginia Tech, Blacksburg, Virginia, United States of America; University of Connecticut School of Medicine, UNITED STATES

## Abstract

For over a century, the Michaelis–Menten (MM) rate law has been used to describe the rates of enzyme-catalyzed reactions and gene expression. Despite the ubiquity of the MM rate law, it accurately captures the dynamics of underlying biochemical reactions only so long as it is applied under the right condition, namely, that the substrate is in large excess over the enzyme-substrate complex. Unfortunately, in circumstances where its validity condition is not satisfied, especially so in protein interaction networks, the MM rate law has frequently been misused. In this review, we illustrate how inappropriate use of the MM rate law distorts the dynamics of the system, provides mistaken estimates of parameter values, and makes false predictions of dynamical features such as ultrasensitivity, bistability, and oscillations. We describe how these problems can be resolved with a slightly modified form of the MM rate law, based on the total quasi-steady state approximation (tQSSA). Furthermore, we show that the tQSSA can be used for accurate stochastic simulations at a lower computational cost than using the full set of mass-action rate laws. This review describes how to use quasi-steady state approximations in the right context, to prevent drawing erroneous conclusions from in silico simulations.

## Introduction

The Michaelis–Menten (MM) rate law has been the dominant paradigm for describing the rates of enzyme-catalyzed reactions for 100 years [1–4]. While the MM rate law was proposed in the pioneering works of Henri [1] and of Michaelis and Menten [2], it was Briggs and Haldane [3] who derived the MM rate law from the underlying molecular mechanism by an approach known as the steady-state approximation. Since then, steady-state approximations have been used to describe other bimolecular interactions, such as reversible binding between a gene and a transcription factor (TF) [5,6] and between a receptor and a ligand [7,8]. In this paper, we refer to the Briggs–Haldane approach as the “standard” quasi-steady state approximation (sQSSA).

Much later, Segel and Slemrod [9] presented a thorough mathematical analysis of the timescale separation that underlies the Briggs–Haldane derivation of the MM rate law. They concluded that the MM rate law is accurate only when the enzyme concentration is low enough so that the concentration of enzyme-substrate complex is negligible compared to substrate concentration [9]. This constraint is acceptable for most metabolic reactions in living cells, where substrate concentrations are typically much larger than the concentrations of enzymes that catalyze the reactions of intermediary metabolism. However, for protein interaction networks that govern many aspects of cell physiology, the “enzymes” and “substrates” are proteins (kinases, phosphatases, TFs, stoichiometric inhibitors, etc.) that are typically present in cells at comparable concentrations [10–13]. Therefore, it is at least suspicious and at worst misleading to use the MM rate law to model enzyme-catalyzed reactions in protein interaction networks. Unfortunately, even in such situations outside its range of validity, the MM rate law has frequently been (mis)used to model enzyme-catalyzed reactions.

In this review, we illustrate how such unjustified use of the MM rate law for protein interaction networks leads to erroneous conclusions. We describe how such errors can often be prevented by replacing the MM rate law with a slightly modified form, based on the “total” quasi-steady state approximation (tQSSA), which is generally accurate for any combination of substrate and enzyme concentrations [14–21]. Specifically, when enzyme concentration is not low enough for enzyme-substrate complex to be negligible compared to the substrate, the tQSSA (but not the MM rate law) can lead to accurate estimation of enzyme kinetic parameters. When the MM rate law is used, zero-order ultrasensitivity and bistability are predicted in cases when they are impossible, but the tQSSA gets it right. Furthermore, a model of a transcriptional negative feedback loop, based on the sQSSA, predicts “no oscillations,” but the same model, based on the tQSSA, is consistent with oscillatory dynamics. Finally, we illustrate that the tQSSA can accurately capture stochastic dynamics of enzyme kinetics, zero-order ultrasensitivity, and oscillations.

This review is written specifically for computational biologists who build, analyze, and simulate mathematical models (deterministic and/or stochastic) of protein interaction networks, to warn them of the dangers of misusing the MM rate law and to provide a simple fix, based on the tQSSA. Our comparisons of sQSSA and tQSSA models are mainly based on simulations. For readers who are more interested in the experimental consequences or the mathematical/statistical properties of sQSSA versus tQSSA, we provide some references to the literature on these topics.

## Results

### Quasi-steady state approximation for enzyme-catalyzed reactions

**Fig 1:**
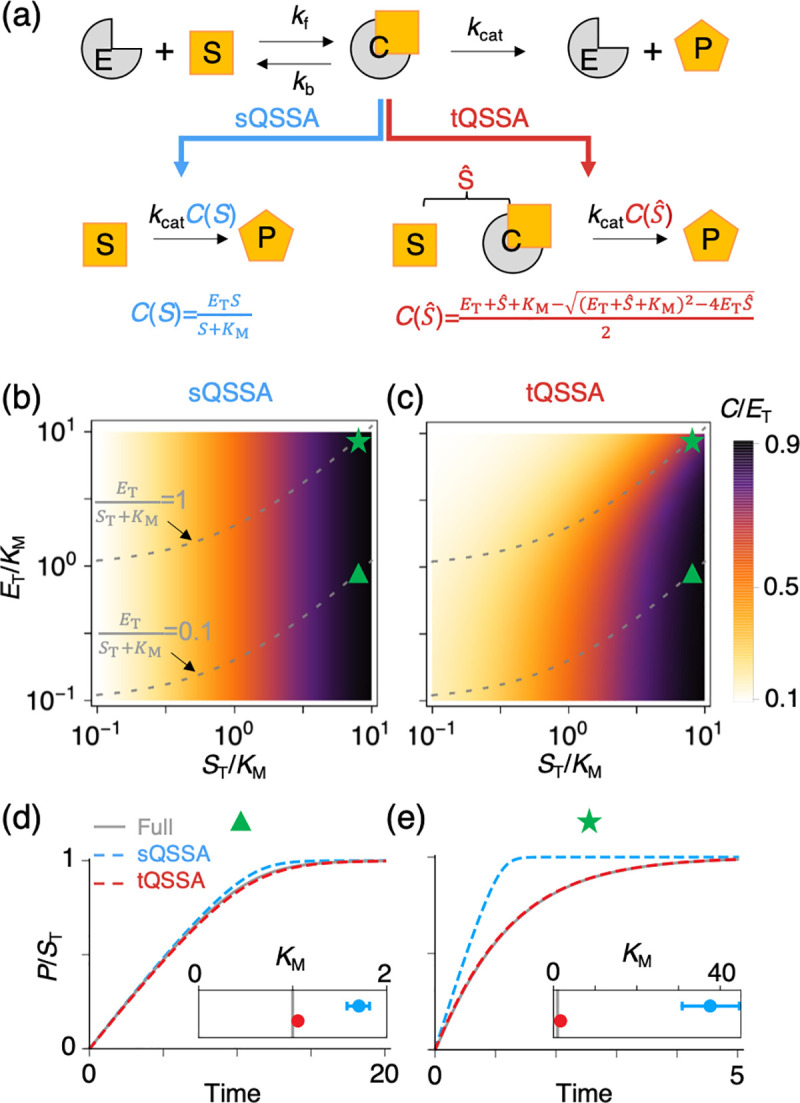
Comparison of the sQSSA and tQSSA for an enzyme-catalyzed reaction. **(a)** The MM mechanism for a single-substrate enzyme-catalyzed reaction; S, substrate; E, enzyme; C, enzyme-substrate complex; P, product. Note that we use a different font style to distinguish a substance, S, from its concentration, *S* in Eq 1. The full model, based on mass-action kinetics (Eq 1), can be simplified by either the sQSSA (Eq 3) or the tQSSA (Eq 5). Both approximations assume that *C* rapidly reaches a QSSA, which is solely determined by either *S* (Eq 2) or $\hat{S}=S+C$ (Eq 4). **(b–c)** Heat maps of the predicted maximal fraction of total enzyme that is in complex with substrate, i.e., *C*(*S*_T_)/*E*_T_, predicted with sQSSA (Eq 2) and tQSSA (Eq 4). In these panels, color represents the ratio *C*(*S*_T_)/*E*_T_, and gray dashed lines are contours of *E*_T_ /(*K*_M_ + *S*_T_) = 0.1 and 1.0. The sQSSA (Eq 2) predicts that this fraction is independent of total enzyme concentration, *E*_T_ (e.g., *C*(*S*_T_)/*E*_T_ = 0.5 when *S*_T_ = *K*_M_ regardless of *E*_T_) in panel b. On the other hand, the tQSSA (Eq 4) predicts that, as *E*_T_ increases, more substrate is needed to achieve that same level of substrate saturation of enzyme molecules in panel c. When the validity condition for the sQSSA holds (i.e., below the gray dashed line where *E*_T_ /(*K*_M_ + *S*_T_) ≪1), the predictions of the two models are nearly identical. **(d–e)** The simulated accumulation of *P* and the estimation of the Michaelis constant, *K*_M_, from the progress curve of *P*. For these calculations, we chose *k*_f_ = 10, *k*_b_ = 9, *k*_cat_ = 1, *K*_M_ = 1, *S*_T_ = 9, and *E*_T_ /(*K*_M_ + *S*_T_) = 0.1 (panel d; triangle mark in (b) and (c)) or 1 (panel e; star mark in (b) and (c)). Initial conditions are *S*(0) = *S*_T_, *C*(0) = 0, *P*(0) = 0. If *E*_T_ is low (i.e., *E*_T_ /(*K*_M_ + *S*_T_) = 0.1), the simulation of *P* and the estimation of *K*_M_ with the sQSSA model are accurate (d). On the other hand, if *E*_T_ is high (i.e., *E*_T_ /(*K*_M_ + *S*_T_) = 1), the simulated *P* with the sQSSA model using the true rate constants is wildly divergent from the true progress curve simulated with the full model (e). As a result, if *k*_cat_ and *K*_M_ are adjusted to fit the sQSSA model to the true progress curve, then the estimates of *k*_cat_ and *K*_M_ are far from the true values (inset; the estimates of *k*_cat_ is not shown). On the other hand, the tQSSA model accurately reproduces the progress curve of *P* and estimates the rate constants regardless of the value of *E*_T_ (d and e). Inset: gray lines are the true value of *K*_M_, and the red and blue circles are the mean *K*_M_ estimates from the tQSSA and sQSSA models, respectively. The bars represent 99% confidence intervals. The Quasi-Newton method is applied to 21 sampled points of the progress curve of *P* simulated with the full model to identify the value of *K*_M_ that minimizes the fitting error of the sQSSA and tQSSA models. The true values of parameters are used as the initial condition for the fitting procedure in order to avoid parameter unidentifiability. MM, Michaelis–Menten; sQSSA, standard quasi-steady state approximation; tQSSA, total quasi-steady state approximation.

A single-substrate enzyme-catalyzed reaction (Fig 1a) can be described by the following system of ordinary differential equations (ODEs) based on mass-action kinetics:

$$\begin{array}{l} \frac{dS}{dt}=-k_{f}S∙E+k_{b}C,\\ \frac{dE}{dt}=-k_{f}S∙E+k_{b}C+k_{cat}C,\\ \frac{dC}{dt}=k_{f}S∙E-k_{b}C-k_{cat}C,\\ \frac{dP}{dt}=k_{cat}C, \end{array}$$ (1)

where *S*, *E*, *C*, and *P* are the time-dependent concentrations of substrate (S), enzyme (E), enzyme-substrate complex (C), and product (P), respectively. Note that we use a different font style to distinguish the name of a substance from its concentration throughout this review. Because $\frac{dE}{dt}=-\frac{dC}{dt},$ the total enzyme concentration (*E*_T_ ≡ *C*+*E*) is constant, as it should be. Furthermore, because the total concentration of substrate + product (*S*_T_ ≡ *S*+*C*+*P*) is also constant, Eq 1 describes the dynamics of only two independent variables, say *C* and *P*. This two-variable model can be simplified to a single-variable model by a “quasi-steady state” approximation (QSSA), which eliminates *C* under the assumption that *C* rapidly reaches a quasi-steady state (QSS) where $\frac{dC}{dt}\approx 0$, and thereafter *C* slowly tracks along the QSS, often referred to as the “slow manifold.” A good approximation to the QSS (i.e., the QSSA) can be obtained by solving $\frac{dC}{dt}=0$:

$$C(S)=\frac{E_{T}S}{S+K_{M}},$$ (2)

where $K_{M}=\frac{k_{b}+k_{\mathrm{cat}}}{k_{f}}$ is the “Michaelis constant” of the enzyme. By substituting the QSSA (Eq 2) for *C* in the full model (Eq 1), we can describe the rate of product accumulation as

$$\frac{dP}{dt}=\frac{k_{\mathrm{cat}}E_{T}S}{S+K_{M}},$$ (3)

which is known as the MM rate law (Fig 1a). Of course, because *S* is continually changing with time as the reaction proceeds, *S* needs to be calculated from the conservation condition, *S*_T_ ≡ *S*+C+*P* (see below).

The QSSA (Eq 2) implies that $\frac{C(S)}{E_{T}}=\frac{S}{K_{M}+S}$ and $\frac{C(K_{M})}{E_{T}}=\frac{1}{2}$, i.e., that enzyme molecules are half-saturated with substrate when *S* = *K*_M_, regardless of total enzyme concentration, *E*_T_ (Fig 1b). This seems unlikely because as *E*_T_ increases, more substrate is likely to be needed for half-saturation of enzyme molecules, suggesting that the QSSA is likely to be valid only if *E*_T_ is small enough. Indeed, Segel and Slemrod showed that the QSSA is valid only when *E*_T_ ≪ *S*_T_ +*K*_M_ [9]. Under this condition, $\frac{C(S_{T})}{S_{T}}=\frac{E_{T}}{K_{M}+S_{T}}\ll 1$, implying that the complex is negligible compared to the total amount of substrate. Thus, *C* can be neglected in the expression for total substrate concentration, *S*_T_ ≡ *S*+*C*+*P*, and *S* can be replaced with *S*_T_−*P* in Eq 3, leading to a single-variable model. We refer to this approximation (Eq 2) as the sQSSA [9], and we refer to Eq 3 (the MM rate law) as the sQSSA model of a single-substrate enzyme-catalyzed reaction. The constraint of low enzyme concentration (*E*_T_ ≪ *S*_T_ +*K*_M_), which is required to use the sQSSA, is acceptable for enzyme-catalyzed reactions in intermediary metabolism, where enzyme concentrations are typically 1,000-fold smaller than metabolite (substrate) concentrations.

On the other hand, in protein interaction networks, both enzyme and substrate are proteins whose intracellular concentrations are roughly comparable [10–13]. In this case, because *C* does not reach the sQSSA (Eq 2) on a short timescale compared to changes in *S* [9], we need to analyze the kinetics of the reaction by a different approximation, the tQSSA [14–21]. For the tQSSA, *S* is replaced with the total substrate concentration $\hat{S}\equiv S+C.$ Now, the validity condition for the QSSA is changed from “*C* reaches QSS before *S* changes appreciably” to “*C* reaches QSS before $\hat{S}$ changes appreciably.” This new condition is easier to satisfy than the old condition because, compared to *S*, $\hat{S}$ (the sum of *S* and *C*) is less likely to change substantially until the product (*P*) has accumulated substantially (see [19,22] for detailed timescale analysis). What a brilliant idea “to make *C* the winner of the race to QSS, change its competitor from a rabbit (*S*) to a turtle ($\hat{S}$)”! (Of course, the rabbit (*S*) is as slow as the turtle ($\hat{S}$) when the validity condition for the MM rate law holds, and thus $\hat{S}\approx S$.)

To make this change, we rewrite the mass-action rate expressions (Eq 1) in terms of $\hat{S}$ instead of *S*:

$$\frac{d\hat{S}}{dt}=-k_{\mathrm{cat}}C,$$

$$\frac{dE}{dt}=-k_{f}(\hat{S}-C)(E_{T}-C)+k_{b}C+k_{\mathrm{cat}}C,$$

$$\frac{dC}{dt}=k_{f}(\hat{S}-C)(E_{T}-C)-k_{b}C-k_{\mathrm{cat}}C,$$

$$\frac{dP}{dt}=k_{\mathrm{cat}}C,$$

which has the same underlying molecular mechanism and dynamics as the original model (Eq 1). Then, the tQSSA is derived in terms of $\hat{S}$ (rather than *S*) by solving the quadratic equation for $\frac{dC}{dt}=0$:

$$C(\hat{S})=\frac{1}{2}\left[E_{T}+\hat{S}+K_{M}-\sqrt{{\left(E_{T}+\hat{S}+K_{M}\right)}^{2}-4E_{T}\hat{S}}\right].$$ (4)

$C(\hat{S})$ is not as elegant as the *C*(*S*) derived with sQSSA (Eq 2), but it is just as easy to implement in a computer program.

A validity condition for the tQSSA (Eq 4) was derived by Tzafriri [16]:

$$\varepsilon_{\mathrm{tQ}}\equiv \frac{k_{\mathrm{cat}}}{2{k_{f}S}_{T}}\left(\frac{E_{T}+K_{M}+S_{T}}{\sqrt{{\left(E_{T}+K_{M}+S_{T}\right)}^{2}-4E_{T}S_{T}}}-1\right)\ll 1.$$

Tzafriri also showed that $\varepsilon_{\mathrm{tQ}}(S_{T},E_{T})\leq \varepsilon_{\mathrm{tQ}}(0,K_{M})=\frac{k_{\mathrm{cat}}}{{4(k}_{b}+k_{\mathrm{cat}})}.$ Thus, if *k*_cat_ ≪ *k*_b_, then *ε*_tQ_ ≪ 1 regardless of total enzyme concentration, *E*_T_. Furthermore, Tzafriri also showed that *ε*_tQ_ ≪ 1 if either *S*_T_ or *E*_T_ (or both) are much greater than K_M_. Importantly, because $\varepsilon_{tQ}\leq \frac{k_{\mathrm{cat}}}{{4(k}_{b}+k_{\mathrm{cat}})}$ < 0.25 always holds, the condition *ε*_tQ_ ≪ 1 is not so badly violated for any cases. This indicates that the tQSSA is generally a good approximation for any ratio of enzyme concentration to substrate concentration and for any ratio of timescales, thanks to the simple but amazing idea of “changing *C*’s competitor from *S* to $\hat{S}$.” We refer readers to [22–24] for stricter validity conditions for sQSSA and tQSSA than those presented in this review, conditions that ensure for the long-time validity of the approximation with a rigorous error analysis.

Unlike $C(S)/E_{T},\;C(\hat{S})/E_{T}$ has the reasonable consequence that, as *E*_T_ increases, more substrate is needed to half-saturate the enzyme (cf. Fig 1b and 1c). This can be seen more clearly with the Padé approximant for $C(\hat{S})$:

$$C(\hat{S})\approx \frac{E_{T}\hat{S}}{K_{M}+E_{T}+\hat{S}},$$

which is valid if *C*^2^ ≪ *E*_T_*S*_T_ [10,16,17]. According to the Padé approximant, enzyme molecules are half-saturated with substrate (i.e., $\frac{C(\hat{S})}{E_{T}}=\frac{1}{2}$), when $\hat{S}$ = *K*_M_ + *E*_T_. That is, the total substrate concentration needed for half-saturation of enzyme increases linearly with total enzyme concentration, *E*_T_ (Fig 1c). Furthermore, when the sQSSA is valid (i.e., *E*_T_ ≪ *S*_T_ + *K*_M_), the Padé approximant becomes nearly identical with *C*(*S*) derived with sQSSA (Eq 2). This explains nearly identical values of *C*(*S*) and $C(\hat{S})$ when *E*_T_ ≪ *S*_T_ + *K*_M_ (Fig 1b and 1c, below the gray dashed line where $\frac{E_{T}}{S_{T}+K_{M}}=0.1$).

By substituting $C(\hat{S})$ (Eq 4) into the full model, we can describe the rate of product accumulation in terms of $\hat{S}$ rather than *S*, and replace the MM rate law (Eq 3) by

$$\frac{dP}{dt}=\frac{k_{cat}}{2}\left[K_{M}+E_{T}+\hat{S}-\sqrt{{\left(K_{M}+E_{T}+\hat{S}\right)}^{2}-4E_{T}\hat{S}}\right],$$ (5)

where $\hat{S}=S_{T}-P$ (Fig 1a). Note that the conservation of *S*_T_ is exact in this tQSSA model, unlike the MM rate law (Eq 3), where the complex is neglected in the conservation condition. Reflecting the validity of tQSSA over a wide range of conditions, simulations of the temporal accumulation of product with the tQSSA model (Eq 5) are consistent with simulations of the full model (Eq 1) regardless of enzyme concentration (Fig 1d and 1e). On the other hand, the sQSSA model (Eq 3) is accurate only when enzyme concentration is low enough compared to *S*_T_ + *K*_M_, i.e., when the sQSSA is valid (Fig 1d and 1e).

### Estimating the rate constants of an enzyme-catalyzed reaction

An accurate model is required for accurate estimation of parameters based on model fitting [13,25,26]. Indeed, in the case of low enzyme concentration, both the sQSSA and the tQSSA models provide good estimates of the “true” rate constants when the models are fitted to the progress curve *P*(*t*) simulated with the full model (i.e., a “progress-curve assay”) (Fig 1d inset). However, for the case of high enzyme concentration, only the tQSSA model provides accurate estimates of the rate constants (Fig 1e inset). Here, to avoid potential unidentifiability of the parameters and thus focus on the bias of estimation caused by model inaccuracy, we used the true parameter values as the initial condition of fitting, under the assumption that we have good prior knowledge. However, in the absence of prior knowledge, the validity of the model reduction (i.e., forward problem) does not guarantee the identifiability of the parameter (i.e., inverse problem) [13]. For instance, if model solutions give equally acceptable fits to the constraining experimental data for several different sets of parameter values, then the “true” parameter values are unidentifiable. Indeed, kinetic parameters only can be estimated with the MM rate law under a stricter condition than its validity condition [27]. Although the identifiability of kinetic parameters with the tQSSA has rarely been explored, a recent numerical study used a Bayesian approach to show that the identifiability of kinetic parameters is not hampered by using the seemingly more complex tQSSA model (Eq 5) instead of the MM rate law for a progress-curve assay [26]. Importantly, due to the unbiased estimation afforded by the tQSSA model regardless of enzyme concentrations, an optimal experiment to identify parameters can be easily designed [26]. However, before the tQSSA model can be used to interpret real experimental data, further experiments and mathematical analysis are needed to validate this in silico study.

When the MM rate law is valid, it should be used for parameter estimation (rather than the tQSSA model) because the MM rate law has been extensively investigated and validated in myriad experimental circumstances [13,27,28]. Furthermore, with the “initial-velocity assay” (based on the initial rate of product accumulation), the estimation of kinetic parameters with the MM rate law is straightforward (e.g., *K*_M_ = substrate concentration at which the initial velocity is half-maximal) [13]. Thus, when the MM validity condition holds, there is no need to consider the more complex tQSSA model instead of the MM rate law.

However, the constraint on using the MM rate law, *E*_T_ ≪ *S*_T_ + *K*_M_, cannot always be satisfied even for in vitro experiments, e.g., a low concentration of substrate is required for sensitive QD-FRET-based probes[29–31]. Importantly, in vivo enzyme concentrations can vary extensively inside a cell and are usually much higher than those used experimentally in vitro [11–13,32]. For instance, the enzyme cytochrome P450, which metabolizes many drugs in the liver, is present in concentrations greatly exceeding [drug] + *K*_M_ [33,34]. However, the MM rate law has been used to predict the rate of drug metabolism in liver and thus drug clearance in approximately 60,000 publications [35]. A recent study describes the errors caused by the MM rate law for predicting hepatic drug clearance and shows how the prediction can be improved by the tQSSA model [36]. However, further study is needed for the application of the tQSSA model to in vivo systems where enzyme concentrations might keep changing dynamically [19].

### Ultrasensitivity and bistability

Ultrasensitivity is a critical property of many signaling networks because it amplifies signals in a nonlinear manner that can trigger a switch-like response [37–40]. Here, we illustrate that, when the MM rate law is misused in models of protein interaction networks, the model may falsely predict ultrasensitivity and bistability.

**Fig 2:**
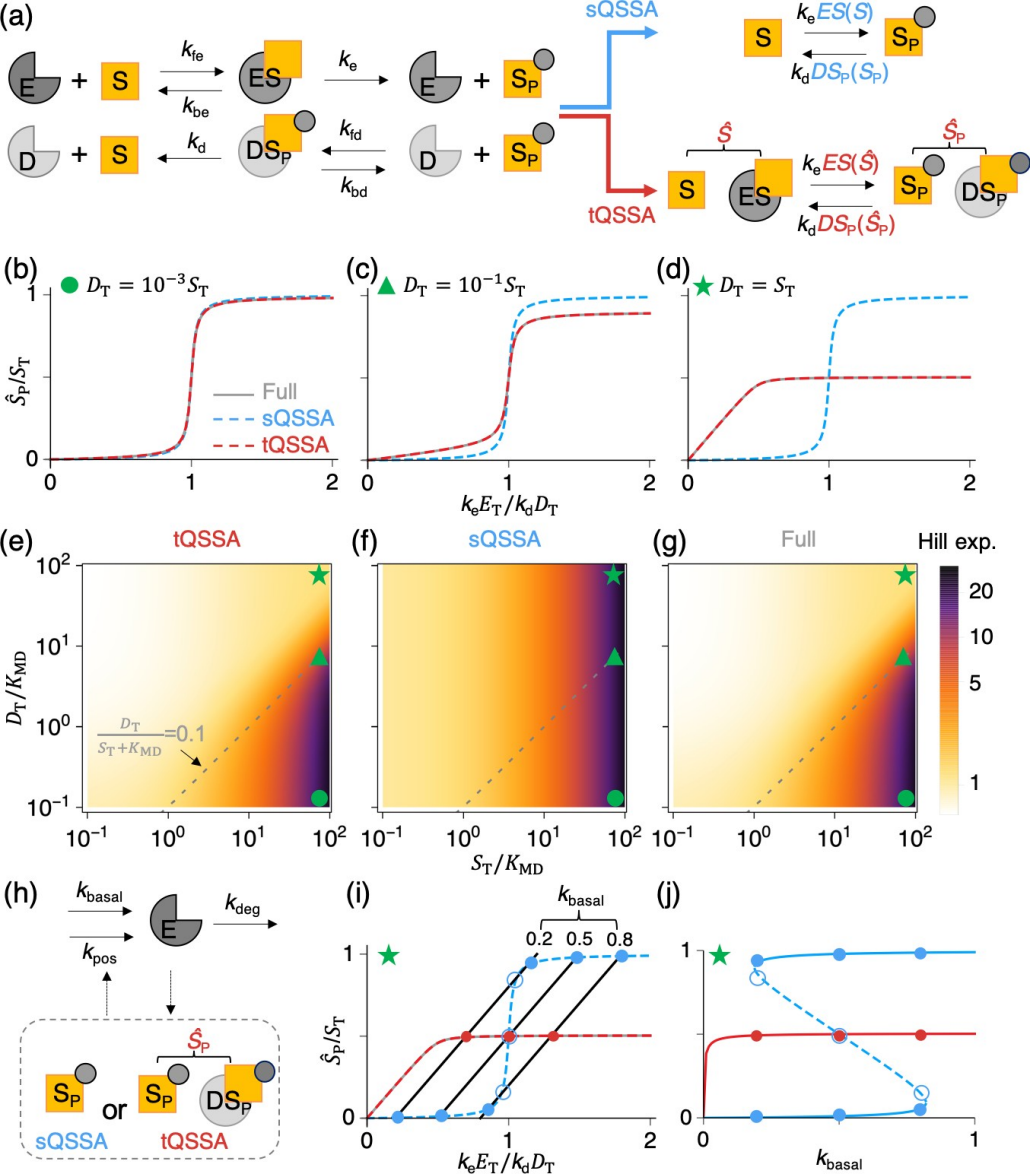
sQSSA, but not tQSSA, makes false predictions of zero-order ultrasensitivity and bistability. **(a)** In the GK mechanism, substrate (S) is phosphorylated by kinase (E), and then phosphorylated substrate (S_P_) is dephosphorylated by phosphatase (D). The full model based on mass-action kinetics (Eq 6) can be simplified by replacing the concentrations of the substrate–kinase complex (*ES*) and phosphorylated substrate–phosphatase complex (*DS*_P_) with either the sQSSA (Eq 7) or the tQSSA (Eq 8). In the tQSSA model, $\hat{S}\equiv S+ES$ and ${\hat{S}}_{P}\equiv S_{P}+DS_{P}$ are total unphosphorylated substrate and phosphorylated substrate concentration, respectively. **(b–d)** As the total concentration of kinase (*E*_T_) increases, the steady-state concentration of phosphorylated substrate (${\hat{S}}_{P}$) increases. The sQSSA model predicts a steep sigmoidal response of ${\hat{S}}_{P}$ regardless of the level of *D*_T_ (in this case, ${\hat{S}}_{P}=S_{P}$, as complexes are assumed to be negligible). On the other hand, both the full and the tQSSA models predict that the steep response is lost as *D*_T_ increases. In these calculations, *S*_T_ = 100, *k*_fe_ = *k*_fd_ = 10, *k*_d_ = *k*_e_ = 1.7, and *K*_ME_ = *K*_MD_ = 1. **(e–g)** Heat maps of the predicted effective Hill exponent of the response function. When the validity of the sQSSA is not satisfied (i.e., above the dashed gray line), the tQSSA model (e) but not the sQSSA model (f) captures the results of the full model (g). Here, the effective Hill exponent is estimated using log[81]/log[EC90/EC10] [42]. The circle, triangle, and star mark represent the parameter values used for (b), (c), and (d), respectively. **(h)** Synthesis and degradation of the kinase (E) are added to the sQSSA and tQSSA models for the GK mechanism (a). In particular, the addition of ${\hat{S}}_{P}$-dependent synthesis of E (*k*_pos_) creates a positive feedback loop between ${\hat{S}}_{P}$ and E. **(i–j)** In panel i, the steady states of the extended positive feedback models (circles) are intersections of the nullclines of *E*_T_ defined by $\frac{dE_{T}}{dt}=0$ (solid black lines) and of the original models of the GK mechanism defined by $\frac{d{\hat{S}}_{P}}{dt}=0$ (colored lines; ${\hat{S}}_{P}=S_{P}$ for the sQSSA model as complexes are assumed to be negligible). As the nullcline of *E*_T_ changes, depending on the basal synthesis rate of E (*k*_basal_), so do the steady states in panel i, which is also illustrated as a bifurcation diagram in panel j. The filled and open circles represent stable and unstable steady states, respectively. In the bifurcation diagram, the solid and dashed lines denote the stable and unstable steady states, respectively. Here, the same parameter set is used as in (d), with *k*_pos_ = *k*_deg_ = $S_{T}^{-1}$ = 0.01. GK, Goldbeter–Koshland; sQSSA, standard quasi-steady state approximation; tQSSA, total quasi-steady state approximation.

In the Goldbeter–Koshland (GK) mechanism of “zero-order ultrasensitivity,” a substrate–product pair (S and S_P_) are interconverted by two enzymes (E and D) (Fig 2a) [41]. This system is commonly observed in phosphorylation and dephosphorylation processes: S is phosphorylated by kinase E, and S_P_ is dephosphorylated by phosphatase D, which can be described by the following system of ODEs:

$$\begin{array}{l} \frac{dS}{dt}=-k_{fe}S∙E+k_{be}ES+k_{d}DS_{P},\\ \frac{dES}{dt}=k_{fe}S∙E-k_{be}ES-k_{e}ES,\\ \frac{dDS_{P}}{dt}=k_{fd}S_{P}∙D-k_{bd}DS_{P}-k_{d}DS_{P}, \end{array}$$ (6)

where concentrations of total substrate (*S*_T_ ≡ *S* + *S*_P_ + *ES* + *DS*_P_), total kinase (*E*_T_ ≡ *E* + *ES*), and total phosphatase (*D*_T_ ≡ *D* + *DS*_P_) are all conserved quantities. As the GK mechanism is composed of two interlocked, single-substrate, enzyme-catalyzed reactions (Fig 1a), it can be simplified by using the QSSA as done in the previous section (Fig 2a).

In particular, assuming low concentrations of total kinase and phosphatase (*E*_T_,*D*_T_ ≪ *S*_T_), Goldbeter and Koshland used the sQSSA for *ES* and *DS*_P_ (Eq 2) to describe the rates of phosphorylation and dephosphorylation in terms of *S* and *S*_P_:

$$\frac{dS_{P}}{dt}=\frac{k_{e}E_{T}S}{S+K_{\mathrm{ME}}}-\frac{k_{d}D_{T}S_{P}}{S_{P}+K_{\mathrm{MD}}},$$ (7)

where $K_{\mathrm{ME}}=\frac{k_{\mathrm{be}}+k_{e}}{k_{\mathrm{fe}}}$ and $K_{\mathrm{MD}}=\frac{k_{\mathrm{bd}}+k_{d}}{k_{\mathrm{fd}}}$ (Fig 2a). Because the sQSSA assumes that *ES* and *DS*_P_ are negligible, they can be neglected in the conservation condition *S*_T_ ≡ *S* + *S*_P_ + *ES* + *DS*_P_, and thus *S* can be replaced with *S*_T_ − *S*_P_ in Eq 7. In this way, Goldbeter and Koshland derived a simple one-variable model, which we refer to as the sQSSA model of the GK mechanism.

Alternatively, the rates of phosphorylation and dephosphorylation can be described using the tQSSA for *ES* and *DS*_P_ (Eq 4) [10,19,25,43,44]. This leads to the tQSSA model of the GK mechanism (Fig 2a):

$$\begin{array}{c} \frac{d{\hat{S}}_{P}}{dt}=\frac{1}{2}k_{e}\left[K_{\mathrm{ME}}+E_{T}+\hat{S}-\sqrt{{\left(K_{\mathrm{ME}}+E_{T}+\hat{S}\right)}^{2}-4E_{T}\hat{S}}\right]\\ -\frac{1}{2}k_{d}\left[K_{\mathrm{MD}}+D_{T}+{\hat{S}}_{P}-\sqrt{{\left(K_{\mathrm{MD}}+D_{T}+{\hat{S}}_{P}\right)}^{2}-4D_{T}{\hat{S}}_{P}}\right], \end{array}$$ (8)

where ${\hat{S}}_{P}\equiv S_{P}+DS_{P}$ and $\hat{S}\equiv S+ES$. Using the conservation condition $S_{T}=(S+ES)+(S_{P}+DS_{P})=\hat{S}+{\hat{S}}_{P},\;\hat{S}$ can be replaced with $S_{T}-{\hat{S}}_{P}$ in the above equation, leading to a one-variable tQSSA model. Note that the complexes (*ES* and *DS*_P_) are not neglected in the conservation condition unlike the sQSSA model.

As the concentration of kinase (*E*_T_) increases, the phosphorylation reaction is more likely to occur, and so the steady-state fraction of phosphorylated substrate (*S*_P_/*S*_T_) increases. For the sQSSA model (Eq 7), Goldbeter and Koshland derived an explicit function for *S*_P_/*S*_T_ in terms of *E*_T_ [10,41]. They showed that for *K*_ME_, *K*_MD_ ≪ *S*_T_, the function is sigmoidally shaped with an abrupt jump from *S*_P_/*S*_T_ ≈ 0 to *S*_P_/*S*_T_ ≈ 1 at *k*_e_*E*_T_ ≈ *k*_d_*D*_T_, i.e., when the maximum rates of phosphorylation and dephosphorylation of substrate are equal (Fig 2b). This behavior is known as “zero-order” ultrasensitivity because the kinase and phosphatase enzymes are—for the most part—saturated with substrate molecules when *K*_ME_, *K*_MD_ ≪ *S*_T_ (i.e., tight binding). A similar sharp transition, close to *k*_e_*E*_T_ ≈ *k*_d_*D*_T_, is also seen for the steady-state fraction of total phosphorylated substrate (${\hat{S}}_{P}/S_{T}$) in the full model (Eq 6) and tQSSA model (Eq 8) when the enzyme concentrations are small compared to total substrate concentration (*E*_T_, *D*_T_ ≪ *S*_T_; Fig 2b and 2e–2g, green circle). On the other hand, they make different predictions as enzyme concentrations increase, and the validity condition for the sQSSA is violated (Fig 2c–2g, green triangle and star). In this case, while both the full and the tQSSA models predict less sensitive responses, the sQSSA model continues to predict (“phantom”) ultrasensitivity.

Embedding an ultrasensitive response in a positive feedback loop is a common mechanism for creating a bistable switch [37,38]; consequently, modeling zero-order ultrasensitivity based on MM rate laws (Eq 7) in a protein interaction network can generate “phantom” bistability as well. To illustrate this, we embed the GK mechanism (Fig 2a) in a positive feedback loop, where both forms of phosphorylated substrate (${\hat{S}}_{P}=S_{P}+{\mathrm{DS}}_{P}$) act as TFs for E, the kinase that converts S to S_P_ (Fig 2h). Specifically, the model of the GK mechanism (Eq 6) is extended to model a positive feedback loop by including the following differential equation for total kinase (*E*_T_):

$$\frac{dE_{T}}{dt}=k_{basal}+k_{pos}{\hat{S}}_{P}-k_{deg}E_{T}.$$ (9)

The extended model of a positive feedback loop can be simplified using either the sQSSA (Eq 7) or the tQSSA (Eq 8), where ${\hat{S}}_{P}=S_{P}$ for the sQSSA model (as *DS*_P_ is assumed to be negligible) or ${\hat{S}}_{P}=S_{P}+DS_{P}$ for the tQSSA model.

The steady states of the sQSSA and tQSSA positive feedback models are found at the intersections of the nullclines of *E*_T_ (Fig 2i, black solid lines) and the models of GK mechanisms (Fig 2i, colored lines for either the full, the sQSSA, or the tQSSA model of the GK mechanism). To illustrate the problem of the sQSSA model, we consider the case of high enzyme concentrations (*D*_T_/*S*_T_ = 1), where the sQSSA and tQSSA models predict different response curves (blue and red) for the GK mechanism (Fig 2d and 2i). Both the tQSSA and the full models have a single stable steady state regardless of *k*_*basal*_ (Fig 2i and 2j, filled red circles). On the other hand, when *k*_basal_ is between 0.19 and 0.82, the sQSSA model predicts two stable states and an unstable state (filled and open blue circles, respectively, in Fig 2i and 2j) due to the “phantom” ultrasensitivity of the sQSSA model (Fig 2d). In this case, the sQSSA model predicts “phantom” bistable behavior, whereas the full and the tQSSA models are monostable.

Such misuse of the MM rate law infected early models of the eukaryotic cell cycle control system proposed by Tyson, Novak, and colleagues [45–48]. They routinely employed sQSSA models of zero-order ultrasensitivity (Eq 7) in their protein interaction networks governing G1/S and G2/M transitions in the cell cycle, and they stressed the idea that bistability plays a crucial role in these transitions. Although the bistable behavior predicted by these models was confirmed in a number of independent experimental studies [49–51], the theoretical foundation of this behavior was shaky because bistability predicted by the sQSSA could not be trusted. Indeed, in later publications, it was shown that bistability is impossible in some of the Novak–Tyson models when the sQSSA is “unpacked” into full mass-action kinetics [52]. Ciliberto and colleagues [10] addressed this problem by comparing sQSSA, tQSSA, and full models of some representative protein interaction networks. First, they considered a simple model of two protein kinases, S and E, that mutually phosphorylate and inactivate each other. They showed that bistability predicted by the sQSSA model is spurious because the full model cannot exhibit bistability for any values of the kinetic rate constants. Bistability can easily be recovered by assuming that S_P_ retains some catalytic activity for phosphorylating E, a fact that is correctly predicted by the tQSSA model. Then, they extended their first simple model to reflect the interactions among the major regulators (MPF, Wee1, and Cdc25) of the G2/M transition in the cell cycle. For biochemically realistic values of the rate constants, their updated models predict bistability, which is reassuring given that this transition is known to be bistable [50,51]; however, time-course curves of the transition from G2 into M simulated by the full model are correctly reproduced by the tQSSA model but not by the sQSSA model [10].

In addition to ultrasensitive responses and bistability, other dynamic properties of signaling networks are more accurately modeled by tQSSA than by sQSSA [19]. For instance, a careful study of phosphorylation and dephosphorylation cycles in the MAP kinase pathway identified various types of input–output characteristics and their noise filtering function with tQSSA, which were not possible with sQSSA [53].

### Biochemical oscillations

To generate periodic oscillations, a negative feedback loop is required [54–58]. For instance, many synthetic oscillators in bacteria are based on transcriptional negative feedback, where a repressor protein directly binds with the promoter of its own gene to suppress its transcription [59–63]. In this case, we can expect that the number of repressor molecules (100s or 1,000s) is in large excess over gene promoter regions (1 or 2) [64,65]. Thus, the repressor–promoter complex is negligible compared to total repressor, and the validity condition for the sQSSA is satisfied. Consequently, MM- or Hill-type functions, derived with the sQSSA, can be used to describe the probability that gene promoter regions are not bound with repressors [66–68].

**Fig 3:**
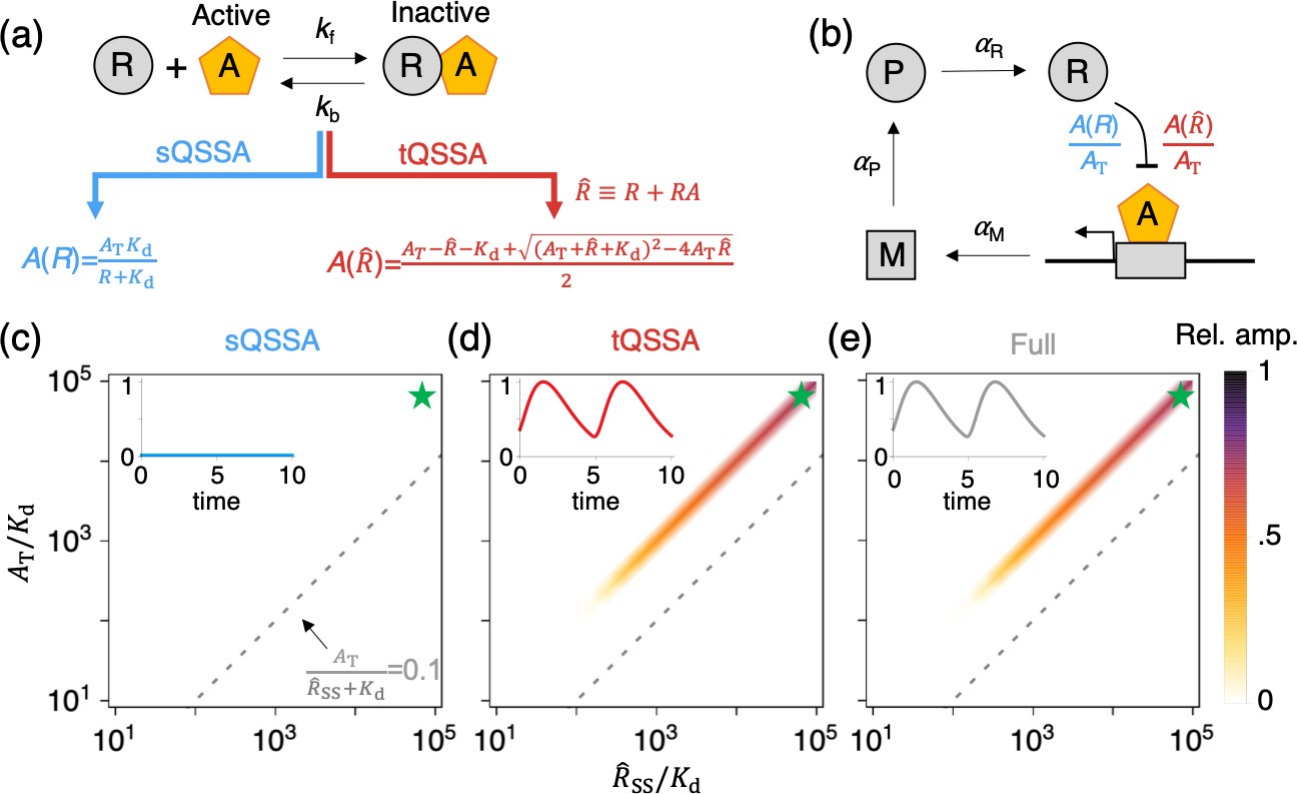
sQSSA, but not tQSSA, leads to the false suppression of oscillations in a protein sequestration–based transcriptional negative feedback loop. **(a)** A repressor protein (R) inhibits its own transcriptional activator (A) by forming a 1:1 stoichiometric complex (RA). The QSS of the free activator, which is not sequestered by the repressor, can be approximated with either the sQSSA or the tQSSA. As the QSSA is derived from reversible binding without any conversion rate, the dissociation constant *K*_d_ = *k*_b_/*k*_f_ is used instead of the Michaelis constant *K*_M_. **(b)** In the transcriptional negative feedback loop, where the repressor protein sequesters the activator protein, the transcription of the repressor’s mRNA is assumed to be proportional to the fraction of total activator that is free (i.e., *A*/*A*_T_), which can be approximated by either the sQSSA or the tQSSA (a). M, mRNA; P, encoded protein; R, repressor protein; A, activator; RA, repressor–activator complex; $\hat{R}$, repressor + repressor–activator complex, A_T_, activator + repressor–activator complex. **(c–e)** Heat maps of the relative amplitude of simulated trajectories of *M*. Oscillations are not possible for the sQSSA model (c). On the other hand, when the validity condition for the sQSSA does not hold (i.e., above the gray dashed line), both the tQSSA (d) and the full (e) models simulate oscillations. In these calculations, *A*_T_ and *α*_M_ are varied so that ${\hat{R}}_{SS}/K_{d}$ (where ${\hat{R}}_{SS}$ is the steady-state value of $\hat{R}$) varies between 10 and 10^5^. Furthermore, *k*_f_ = *k*_b_ = 10 (i.e., *K*_d_ = 1) and all other parameter values = 1. Insets: for a particular choice of parameter values (star mark), trajectories of *M* are simulated, and all trajectories are normalized by the maximum value of the trajectory simulated by the full model. QSS, quasi-steady state; sQSSA, standard quasi-steady state approximation; tQSSA, total quasi-steady state approximation.

In other cases, an intermediate molecule is involved in the transcriptional negative feedback loop [54]. For instance, repression of transcription can occur via protein sequestration [69–71], when a repressor sequesters a transcriptional activator in a 1:1 stoichiometric complex rather than directly binding to its own gene promoter (Fig 3a). To describe the binding of repressor and activator proteins whose concentrations can be comparable, the sQSSA is not appropriate, and the tQSSA should be used.

To illustrate the problem, let’s compare sQSSA and tQSSA models of a protein sequestration–based transcriptional negative feedback loop for mRNA (M), encoded protein (P), and repressor protein (R) (Fig 3b):

$$\frac{dM}{dt}=\alpha_{M}\frac{A(\hat{R})}{A_{T}}-\beta_{M}M,$$

$$\frac{dP}{dt}=\alpha_{P}M-\beta_{P}P,$$

$$\frac{d\hat{R}}{dt}=\alpha_{R}P-\beta_{R}\hat{R},$$

where the ratio of free activator to total activator, $A(\hat{R})/A_{T}$, is to be given by either the sQSSA or the tQSSA, as displayed in Fig 3a. $\hat{R}=R+RA$ for the tQSSA model and $\hat{R}=R$ for the sQSSA model as the complex (*RA*) is assumed to be negligible.

In the sQSSA model, free activator concentration is given by $A(R)=\frac{{A_{T}K}_{d}}{K_{d}+R}$, and so the rate of transcription of mRNA is $\frac{{\alpha_{M}K}_{d}}{K_{d}+R}$ (Fig 3a left). This model is a special case of Goodwin’s classic model of periodic gene expression, where transcriptional suppression is described by a Hill function $\frac{\alpha_{M}K_{d}^{n}}{K_{d}^{n}+R^{n}}$ (i.e., when *n* = 1) [72]. It is well known that Goodwin’s model exhibits oscillations only if *n* > 8 [67,73,74]. That is to say, for the sQSSA model with $\frac{{\alpha_{M}K}_{d}}{K_{d}+R}$, the feedback effect does not have a degree of cooperativity large enough to generate oscillations. Indeed, the sQSSA model cannot produce oscillations for any choice of parameter values (Fig 3c).

The tQSSA model, known as the Kim–Forger model, was developed to describe the circadian clock in mammalian cells [70,71,75,76]. Unlike the sQSSA model, it exhibits sustained oscillation for comparable concentrations of total activator (*A*_T_) and the total repressor ($\hat{R}$), provided *K*_d_ ≪ *A*_T_, i.e., tight binding between them (Fig 3d). The full model, based on mass-action kinetics, exhibits oscillations that are nearly identical to the tQSSA model, provided fast, reversible, tight binding between repressor and activator (Fig 3e). Both the full and the tQSSA models exhibit oscillations in a region of parameter space where the sQSSA model is invalid, i.e., above the gray dashed line in Fig 3d and 3e. On the other hand, all three models agree that oscillations are impossible when activator concentration is low (below the gray dashed line in Fig 3c–3e). Once again, the sQSSA model is accurate when its validity condition holds but can cause errors outside of its range of validity. In this particular case, the sQSSA model destroys the ultrasensitive response of transcription rate to repressor accumulation that is required to generate oscillations [54,58,70,71].

Unlike the sQSSA model, the tQSSA model predicts oscillations when the amounts of activator and repressor are similar, and they bind tightly (Fig 3d). This is the case for the mammalian circadian clock: repression of transcription mainly occurs via protein sequestration [77–79] with a tight binding [80], and the amounts of the key activator (BMAL1) and repressor (PER1/2) are similar [81,82]. Importantly, as their ratio becomes closer to 1:1, the amplitude and stability of circadian rhythms increase [77,83], consistent with predictions of the tQSSA model (Fig 3d) [70,71]. Other properties of the mammalian circadian clock (e.g., robust network design) are also accurately captured by the tQSSA model [71]. Because the amounts of activator and repressor are similar in the mammalian circadian clock, using the sQSSA model would be inappropriate. However, mechanisms for which the sQSSA model would be appropriate, such as incorporation of an additional positive feedback loop or cooperative multisite phosphorylation, have been proposed as key oscillatory mechanisms for the mammalian circadian clock, but experimental evidence for these mechanisms are not presently available (see [71] for details).

### Stochastic total quasi-steady state approximation

The chemical master equation (CME) is a popular way to capture the stochasticity of biochemical reactions. In the presence of timescale separation, the CME can be simplified by assuming that fast species are in QSS. The stochastic QSSA of a fast species is its conditional average number given the numbers of molecules of the slow species [84–87]. However, calculation of the conditional average has been possible so far in very limited circumstances, such as linear reaction kinetics, simple birth–death processes, feed-forward networks, and complex-balanced networks [88–91]. In the hope of making progress in the general case, modelers have approximated the stochastic QSSA with the more easily derived deterministic QSSA obtained from the corresponding deterministic ODE system [92–95]. Specifically, nonelementary reaction rates based on the deterministic QSSA (e.g., the MM function) are converted to stochastic propensity functions by converting the concentration of a species, [X], to its number of molecules, *N*_X_, with the relationship *N*_X_ = [X]∙Ω, where Ω is the volume of the system. Then, based on the nonelementary propensity functions, stochastic simulations are performed with Gillespie’s algorithm [92–95]. In this way, a deterministic model simplified with QSSA can be used to perform stochastic simulations.

**Fig 4:**
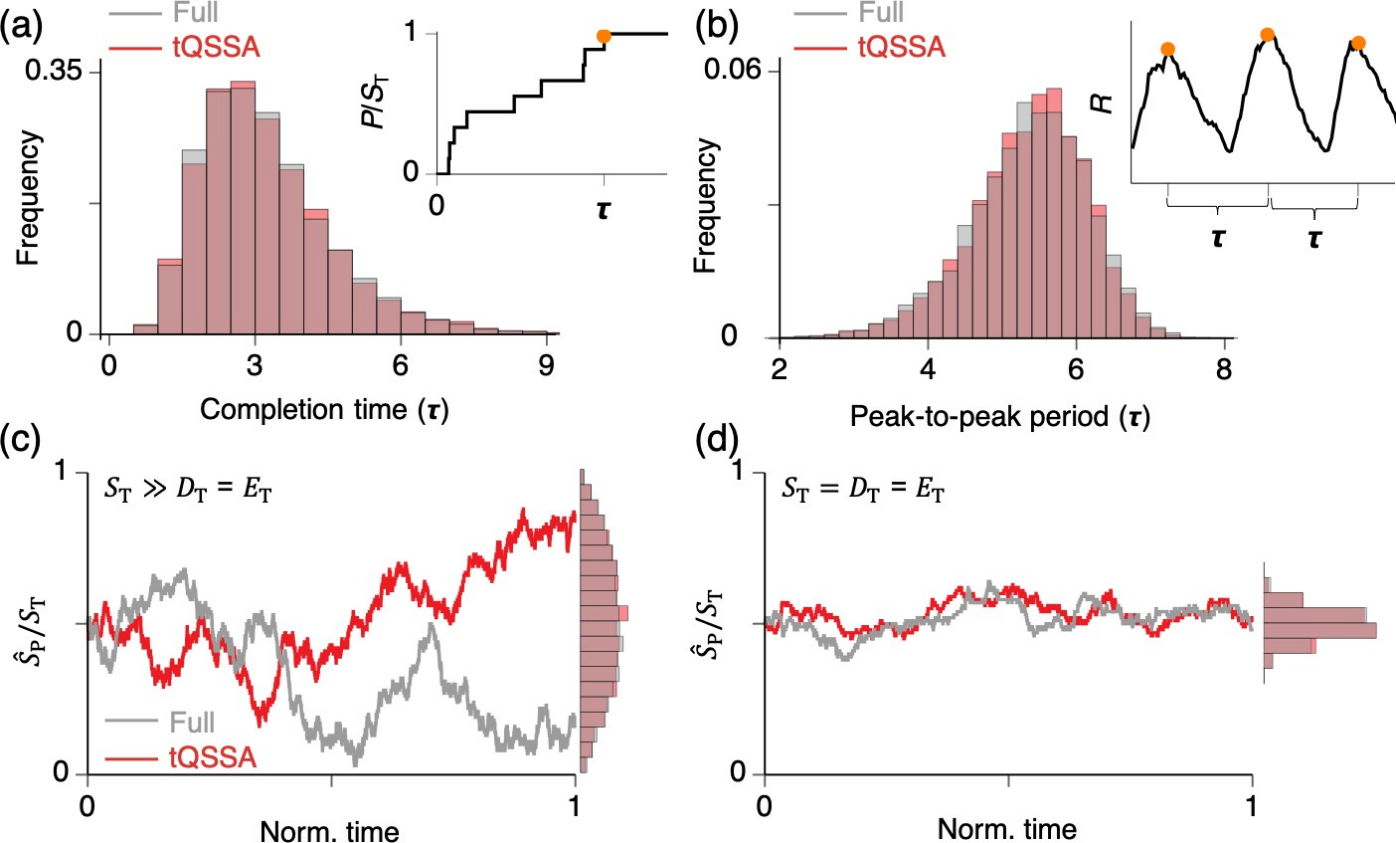
tQSSA provides accurate stochastic simulations. **(a)** Catalysis-completion time τ (see inset) of the single-substrate enzyme-catalyzed reaction (Fig 1a) varies due to stochastic fluctuations. The distributions of τ simulated with the full model (3.24 ± 1.4) and the tQSSA model (3.22 ± 1.37) are nearly identical. Here, the same parameter set and initial condition are used as in Fig 1e and Ω = 1. Gillespie’s stochastic simulation algorithm is used [107]. **(b)** The peak-to-peak period τ (see inset) of simulated oscillatory trajectories for the model of a negative feedback loop (Fig 3b) fluctuates due to stochasticity. The distributions of τ simulated by the full model (5.3 ± 0.8) and tQSSA model (5.3 ± 0.78) are nearly identical. Here, the same parameter set and initial condition are used as in the inset of Fig 3d and 3e and Ω = 5 × 10^−4^. (**c–d)** In both the presence (c) and absence (d) of zero-order ultrasensitivity, stochastic simulations of the full and tQSSA models lead to nearly identical stationary distributions of ${\hat{S}}_{P}/S_{T}$: 0.5 ± 0.23 and 0.5 ± 0.22 (c) and 0.5 ± 0.05 and 0.5 ± 0.05 (d). Here, *E*_T_ = *D*_T_ and the values of the other parameters are the same as used in Fig 2b and 2d for (c) and (d), respectively. Initial conditions are *S*_p_(0) = *S*(0) = *S*_T_/2, *D*(0) = *E*(0) = *D*_T_, and Ω = 1. tQSSA, total quasi-steady state approximation.

This heuristic approach has been widely used in the hope that simulations of the simplified stochastic model will be accurate; however, this is often not the case when the deterministic sQSSA is used to calculate propensity functions [96–99]. On the other hand, when the deterministic tQSSA (e.g., Eq 4) is used, stochastic simulations have been surprisingly accurate with much lower computation cost [26,98–105]. To illustrate, consider first the case of a single-substrate enzyme-catalyzed reaction (Fig 1a). In Fig 4a inset, we simulate the catalysis-completion time τ, i.e., the time elapsed for all of the initial substrate to be converted to product. This time varies from one simulation to another, of course, because of stochastic fluctuations in chemical reactions involving small numbers of molecules. From many realizations of the stochastic process, the distributions of τ for the full and tQSSA models are calculated, and they are nearly identical (Fig 4a). Similarly, fluctuations of the oscillatory period (peak-to-peak duration) of a transcriptional negative feedback loop (Fig 3a) are also accurately captured by the stochastic tQSSA model (Fig 4b). As a third example, simulations performed with the stochastic tQSSA model of the GK mechanism (Fig 2a) are accurate both when *D*_T_/*S*_T_ ≪ 1 (Fig 4c) and when *D*_T_ /*S*_T_ = 1 (Fig 4d). When *D*_T_ /*S*_T_ ≪ 1, the GK mechanism exhibits zero-order ultrasenstivity, i.e., the fraction of total phosphorylated substrate changes sharply around *E*_T_ *= D*_T_ (Fig 2b). Reflecting this sharp change, the fraction of the total phosphorylated substrate (${\hat{S}}_{P}/S_{T}$) fluctuates wildly in the stochastic simulations, and the stationary distribution of ${\hat{S}}_{P}/S_{T}$ is broad (Fig 4c). On the other hand, when *D*_T_/*S*_T_ = 1, the fraction of total phosphorylated substrate changes very little around *E*_T_ *= D*_T_ (Fig 2d), and thus its stationary distribution is narrow (Fig 4d).

The accuracy of the stochastic simulation using the deterministic tQSSA stems from the accuracy of the deterministic tQSSA over a wide range of conditions [99]. In the presence of timescale separation, a deterministic solution evolves in two phases: an initial transient phase and a QSS phase. The two phases correspond, respectively, to times before and after the solution is in QSS (i.e., “on the slow manifold”). During an initial transient phase, the fast variable rapidly reaches the slow manifold. Then, the system evolves along the slow manifold during the QSS phase. By contrast, the trajectory of the stochastic system, unlike the trajectory of the deterministic system, keeps jumping off the slow manifold due to fluctuations. Thus, unlike the deterministic system, transient phases continually recur whenever the stochastic system leaves the slow manifold due to a fluctuation. Importantly, depending on exactly how the trajectory escapes the slow manifold, the transient phase will begin from different initial conditions. Thus, for accurate stochastic simulations, the deterministic QSSA must be accurate not only for specific initial conditions but also for a range of initial conditions that cover the most likely fluctuations away from the slow manifold. For this reason, the tQSSA is a better choice than the sQSSA for stochastic simulations, because the tQSSA is accurate over a much wider range of initial conditions than the sQSSA (see [99] for details).

However, in the presence of large fluctuations, the moment closure assumption underlying the replacement of the stochastic QSSA with the deterministic tQSSA [98,103,105] can cause considerable errors [90]. In this case, model reduction based on exact moment derivation without the moment closure assumption is needed [90], or a recently developed approximation based on curvature of the slow manifold and initial transient flow field can be used [106]. See [87] for the other types of approximations for CMEs with timescale separation. If all else fails, the full system of mass-action rate laws can be simulated by Gillespie’s stochastic simulation algorithm.

## Conclusion

The MM rate law has been a great asset in describing enzyme-catalyzed reactions for more than a century. It is valid for most metabolic reactions in living cells, where substrate concentrations are much larger than the concentrations of enzymes that catalyze the reactions of intermediary metabolism. However, when used outside of its domain of validity (low enzyme concentrations), the MM rate law can cause serious errors of dynamics, thereby reducing the reliability of MM-based models (Figs 1–3). Of most relevance in this regard are protein interaction networks, for which enzymes and their substrates are usually present in comparable concentrations; hence, the validity condition for the MM rate law is in doubt, and the conclusions of models based on the MM rate laws are seriously compromised.

It is often difficult to check the validity condition (*E*_T_ ≪ *S*_T_ + *K*_M_) of the MM rate law due to limited information about the underlying system. For instance, it is challenging to measure in vivo enzyme concentrations, which might be highly heterogeneous across cell types. For the case of gene regulation by TF binding to gene regulatory sequences, an MM-type rate law is likely to be valid because TFs are usually in large excess over their chromosome-binding sites [64,65]. However, when intermediate molecules are involved, such as cofactors that mediate the interactions between TFs and gene promoter regions, it is risky to use an MM rate law (a hyperbolic function for the probability of gene transcription) to describe such gene regulatory networks (Fig 3). Similarly, it is risky to use an MM-type function to describe the interaction between ligands and receptors, when their concentrations are comparable [8]. In all these situations, we need an alternative to MM-type rate laws.

Classically, the MM rate law was derived by Briggs and Haldane [3] by assuming that the enzyme-substrate complex rapidly reaches a quasi-steady state concentration determined by the substrate concentration. Their “standard” quasi-steady state approximation (sQSSA) is valid if the enzyme concentration is low enough so that the concentration of enzyme-substrate complex is negligible compared to substrate concentration. In cases where this proviso is not satisfied, there is a simple fix: to re-derive the rate law based on the “total” quasi-steady state approximation (tQSSA). The tQSSA is better than the sQSSA because it is accurate for a much wider range of conditions, as exemplified in Figs 1–3. Furthermore, the tQSSA is accurate even for stochastic simulations (Fig 4). Admittedly, the tQSSA rate law (Eq 4) is less intuitively appealing than the MM rate law (Eq 2) and less amenable to mathematical analysis, but for computer simulations, there is no drawback to using the tQSSA in place of an MM rate law.

In light of the uncertainties surrounding sQSSA models, why has this approach been so popular among modelers, including (sorry to say) the senior author of this review? Well, the MM rate law is well known and respected by molecular cell biologists, so, of course, it seemed like a good place to start. Signaling pathways often exhibit highly sigmoidal responses [38], and “zero-order ultrasensitivity” (which is based on the MM rate law) is a simple and elegant way to model such behavior. When problems with the sQSSA approach were recognized, the suspicious models were justified as “phenomenological” rather than “mechanistic.” As a last resort, one could always fall back on the “spherical cow” defense, i.e., it’s OK to make unrealistic assumptions if they capture the “relevant details” of the problem.

Indeed, it is a common and respectable practice to simplify complex systems by neglecting irrelevant details. However, according to Kruskal’s minimum simplification principle [108], “no term should be neglected without a good reason.” When the sQSSA is used to simplify a complex reaction mechanism without good reason (e.g., when its validity condition is not satisfied), the resulting model can cause errors of both steady-state and transient behaviors [109–112]. In particular, when the MM rate law is used outside its domain of validity, the combination of faulty QSS assumptions and neglect of enzyme-substrate complexes causes serious errors of dynamics, thereby reducing the reliability of MM-based models, as illustrated in Figs 1–3.

One must bear in mind that the tQSSA also has validity constraints: it is invalid if *k*_cat_ ≫ *k*_b_ and *E*_T_ + *S*_T_ is not in excess over *K*_M_ [16,24]. These constraints are not likely to limit tQSSA’s applicability to protein interaction networks, but they must be checked if we are to take Kruskal’s principle seriously.

In light of the uncertainties involved in any QSSA, why not use the full mass-action kinetic equations (e.g., Eq 1)? Because the QSSA reduces the dimension of a dynamical system, the simplified system of equations can often be analyzed more readily than the full set of equations. For example, the phase plane portrait of the reduced model in Fig 2i provides real insight into the properties of the full, six-variable model of a bistable switch. Furthermore, both sQSSA and tQSSA models have fewer kinetic parameters than the full model, which allows us to sidestep what might be serious parameter identifiability problems [26,27]; in particular, the forward and reverse binding constants (*k*_f_ and *k*_b_) of the enzyme-substrate complex disappear in the reduced models. Hence, it is not necessary to measure experimentally the rate constants of these (presumably) fast reactions, only their ratio, $K_{M}=\frac{k_{b}+k_{\mathrm{cat}}}{k_{f}}\approx \frac{k_{b}}{k_{f}}$, matters, and *K*_M_ can often be estimated experimentally (e.g., [80]).

Because the simple systems that we have considered in this review are often used as modules in more complex reaction mechanisms, the tQSSA can be used to simplify these complex systems [10,19,25,113–116]. When doing so, as multiple timescales may exist, the validity conditions for the tQSSA should be carefully checked so that the tQSSA is not misused. For instance, the rates of association or dissociation of complexes should be fast compared to other rate processes in the network, such as synthesis, degradation, phosphorylation, and dephosphorylation. Furthermore, unlike the examples considered in this review, the algebraic equations underlying the tQSSA of more complex systems may share variables in ways that preclude explicit solution [10], and the QSS equations need to be solved numerically. Excellent computer algorithms for solving differential-algebraic equations are available in [117].

In circumstances where a tQSSA model may be preferred over an sQSSA model, the problem lies in identifying the proper change from fast variables to slower ones (i.e., rabbits to turtles) that ensures a separation of timescales over a wide range of conditions [53,113]. For instance, in enzyme kinetics, the concentration of free substrate, *S*, was replaced with the slower variable, $\hat{S}$ (Fig 1a), and in the GK mechanism, *S* and *S*_P_ were replaced with the slower variables $\hat{S}$ and ${\hat{S}}_{P}$ (Fig 2a). For complicated reaction networks, the appropriate change of variables can be hard to see [113]. Thus, it would be nice to have an algorithm that identifies an optimal change of variables and performs symbolic or numerical calculation of QSS equations. Such facilities would greatly improve the reliability of dynamical models in computational cell biology.

Taking all these factors into consideration, we recommend to computational biologists who are modeling protein interaction networks with disparate timescales that they simplify their models using the tQSSA rather than the sQSSA. In situations where binding partners (e.g., enzyme and substrate, receptor and ligand, and transcriptional regulators) are in comparable concentrations, tQSSA models are more reliable in predicting the dynamical properties of deterministic interactions and in capturing the fluctuations of stochastic interactions.

## References

[1] Henri V. Lois générales de l’action des diastases. Librairie Scientifique A. Hermann; 1903.

[2] Menten L, Michaelis M. Die kinetik der invertinwirkung. Biochem Z. 1913;49(333–369):5.

[3] Briggs GE, Haldane JBS. A note on the kinetics of enzyme action. Biochem J. 1925;19(2):338 10.1042/bj0190338 16743508 PMC1259181

[4] Gunawardena J. Time-scale separation—Michaelis and Menten’s old idea, still bearing fruit. FEBS J. 2014;281(2):473–488. 10.1111/febs.12532 24103070 PMC3991559

[5] Bolouri H, Davidson EH. Modeling transcriptional regulatory networks. Bioessays. 2002;24(12):1118–1129. 10.1002/bies.10189 12447977

[6] Rué P, Garcia-Ojalvo J. Modeling gene expression in time and space. Annu Rev Biophys. 2013;42:605–627. 10.1146/annurev-biophys-083012-130335 23527779

[7] Attie AD, Raines RT. Analysis of receptor–ligand interactions. J Chem Educ. 1995;72(2):119 10.1021/ed072p119 28736457 PMC5521016

[8] Pollard TD. A guide to simple and informative binding assays. Mol Biol Cell. 2010;21(23):4061–4067. 10.1091/mbc.E10-08-0683 21115850 PMC2993736

[9] Segel LA, Slemrod M. The quasi-steady-state assumption: a case study in perturbation. SIAM Rev. 1989;31(3):446–477.

[10] Ciliberto A, Capuani F, Tyson JJ. Modeling networks of coupled enzymatic reactions using the total quasi-steady state approximation. PLoS Comput Biol. 2007;3(3):e45 10.1371/journal.pcbi.0030045 17367203 PMC1828705

[11] Fujioka A, Terai K, Itoh RE, Aoki K, Nakamura T, Kuroda S, et al Dynamics of the Ras/ERK MAPK cascade as monitored by fluorescent probes. J Biol Chem. 2006;281(13):8917–8926. 10.1074/jbc.M509344200 16418172

[12] Blüthgen N, Bruggeman FJ, Legewie S, Herzel H, Westerhoff HV, Kholodenko BN. Effects of sequestration on signal transduction cascades. FEBS J. 2006;273(5):895–906. 10.1111/j.1742-4658.2006.05105.x 16478465

[13] Schnell S, Maini P. A century of enzyme kinetics: reliability of the KM and vmax estimates. Comm Theor Biol. 2003;8:169–187.

[14] Cha S. Kinetic behavior at high enzyme concentrations magnitude of errors of Michaelis–Menten and other approximations. J Biol Chem. 1970;245(18):4814–4818. 5456154

[15] Laidler KJ. Theory of the transient phase in kinetics, with special reference to enzyme systems. Can J Chem. 1955;33(10):1614–1624.

[16] Tzafriri AR. Michaelis–Menten kinetics at high enzyme concentrations. Bull Math Biol. 2003;65(6):1111–1129. 10.1016/S0092-8240(03)00059-4 14607291

[17] Borghans JA, De Boer RJ, Segel LA. Extending the quasi-steady state approximation by changing variables. Bull Math Biol. 1996;58(1):43–63. 10.1007/BF02458281 8819753

[18] Schnell S, Maini PK. Enzyme kinetics far from the standard quasi-steady-state and equilibrium approximations. Math Comput Model. 2002;35(1–2):137–144.

[19] Bersani AM, Bersani E, Dell’Acqua G, Pedersen MG. New trends and perspectives in nonlinear intracellular dynamics: one century from Michaelis–Menten paper. Contin Mech Thermodyn. 2015;27(4–5):659–684.

[20] Tzafriri AR, Edelman ER. Quasi-steady-state kinetics at enzyme and substrate concentrations in excess of the Michaelis–Menten constant. J Theor Biol. 2007;245(4):737–748. 10.1016/j.jtbi.2006.12.005 17234216

[21] Lim HC. On kinetic behavior at high enzyme concentrations. AICHE J. 1973;19(3):659–661.

[22] Eilertsen J, Schnell S. The quasi-state-state approximations revisited: timescales, small parameters, singularities, and normal forms in enzyme kinetics. Math Biosci. 2020;325:108339 10.1016/j.mbs.2020.108339 32184091 PMC7337988

[23] Goeke A, Walcher S, Zerz E. Determining “small parameters” for quasi-steady state. J Differ Equ. 2015;259(3):1149–1180.

[24] Patsatzis DG, Goussis DA. A new Michaelis–Menten equation valid everywhere multi-scale dynamics prevails. Math Biosci. 2019;315:108220 10.1016/j.mbs.2019.108220 31255632

[25] Pedersen MG, Bersani AM, Bersani E, Cortese G. The total quasi-steady-state approximation for complex enzyme reactions. Math Comput Simul. 2008;79(4):1010–1019.

[26] Choi B, Rempala GA, Kim JK. Beyond the Michaelis–Menten equation: accurate and efficient estimation of enzyme kinetic parameters. Sci Rep. 2017;7(1):1–11. 10.1038/s41598-016-0028-x 29208922 PMC5717222

[27] Stroberg W, Schnell S. On the estimation errors of KM and V from time-course experiments using the Michaelis–Menten equation. Biophys Chem. 2016;219:17–27. 10.1016/j.bpc.2016.09.004 27677118

[28] Yun K-I, Han T-S. Relationship between enzyme concentration and Michaelis constant in enzyme assays. Biochimie. 2020. 10.1016/j.biochi.2020.06.002 32585228

[29] Algar WR, Malanoski AP, Susumu K, Stewart MH, Hildebrandt N, Medintz IL. Multiplexed tracking of protease activity using a single color of quantum dot vector and a time-gated Forster resonance energy transfer relay. Anal Chem. 2012;84(22):10136–10146. 10.1021/ac3028068 23128345

[30] Sapsford KE, Farrell D, Sun S, Rasooly A, Mattoussi H, Medintz IL. Monitoring of enzymatic proteolysis on a electroluminescent-CCD microchip platform using quantum dot-peptide substrates. Sensors Actuators B Chem. 2009;139(1):13–21.

[31] Algar WR, Malonoski A, Deschamps JR, Blanco-Canosa JB, Susumu K, Stewart MH, et al Proteolytic activity at quantum dot-conjugates: kinetic analysis reveals enhanced enzyme activity and localized interfacial “hopping”. Nano Lett. 2012;12(7):3793–3802. 10.1021/nl301727k 22731798 PMC9354701

[32] Albe KR, Butler MH, Wright BE. Cellular concentrations of enzymes and their substrates. J Theor Biol. 1990;143(2):163–195. 10.1016/s0022-5193(05)80266-8 2200929

[33] Houston JB, Kenworthy KE. In vitro–in vivo scaling of CYP kinetic data not consistent with the classical Michaelis–Menten model. Drug Metab Dispos. 2000;28(3):246–254. 10681367

[34] Wienkers LC, Heath TG. Predicting in vivo drug interactions from in vitro drug discovery data. Nat Rev Drug Discov. 2005;4(10):825 10.1038/nrd1851 16224454

[35] Benet L, Liu S, Wolfe A. The universally unrecognized assumption in predicting drug clearance and organ extraction ratio. Clin Pharmacol Ther. 2018;103(3):521–525. 10.1002/cpt.802 28762489 PMC6364827

[36] Hm B, Hy Y, Kim SK, Kim JK. Beyond the Michaelis–Menten: accurate prediction of in vivo hepatic clearance for drugs with low KM. Clin Transl Sci. 2020. 10.1111/cts.12804 PMC7719389 32324332

[37] Tyson JJ, Chen KC, Novak B. Sniffers, buzzers, toggles and blinkers: dynamics of regulatory and signaling pathways in the cell. Curr Opin Cell Biol. 2003;15(2):221–231. 10.1016/s0955-0674(03)00017-6 12648679

[38] Ferrell JE Jr, Ha SH. Ultrasensitivity part I: Michaelian responses and zero-order ultrasensitivity. Trends Biochem Sci. 2014;39(10):496–503. 10.1016/j.tibs.2014.08.003 25240485 PMC4214216

[39] Haney S, Bardwell L, Nie Q. Ultrasensitive responses and specificity in cell signaling. BMC Syst Biol. 2010;4(1):119. 20735856 10.1186/1752-0509-4-119 PMC2940771

[40] Shis DL, Bennett MR, Igoshin OA. Dynamics of bacterial gene regulatory networks. Annu Rev Biophys. 2018;47:447–467. 10.1146/annurev-biophys-070317-032947 29570353

[41] Goldbeter A, Koshland DE. An amplified sensitivity arising from covalent modification in biological systems. Proc Natl Acad Sci U S A. 1981;78(11):6840–6844. 10.1073/pnas.78.11.6840 6947258 PMC349147

[42] Ha S, Ferrell J. Thresholds and ultrasensitivity from negative cooperativity. Science. 2016;352(6288):990–993. 10.1126/science.aad5937 27174675 PMC5184821

[43] Straube R. Operating regimes of covalent modification cycles at high enzyme concentrations. J Theor Biol. 2017;431:39–48. 10.1016/j.jtbi.2017.08.006 28782551

[44] Pedersen MG, Bersani AM. Introducing total substrates simplifies theoretical analysis at non-negligible enzyme concentrations: pseudo first-order kinetics and the loss of zero-order ultrasensitivity. J Math Biol. 2010;60(2):267–283. 10.1007/s00285-009-0267-6 19333602

[45] Chen KC, Csikasz-Nagy A, Gyorffy B, Val J, Novak B, Tyson JJ. Kinetic analysis of a molecular model of the budding yeast cell cycle. Mol Biol Cell. 2000;11(1):369–391. 10.1091/mbc.11.1.369 10637314 PMC14780

[46] Marlovits G, Tyson CJ, Novak B, Tyson JJ. Modeling M-phase control in Xenopus oocyte extracts: the surveillance mechanism for unreplicated DNA. Biophys Chem. 1998;72(1–2):169–184. 10.1016/s0301-4622(98)00132-x 9652093

[47] Novak B, Csikasz-Nagy A, Gyorffy B, Chen K, Tyson JJ. Mathematical model of the fission yeast cell cycle with checkpoint controls at the G1/S, G2/M and metaphase/anaphase transitions. Biophys Chem. 1998;72(1–2):185–200. 10.1016/s0301-4622(98)00133-1 9652094

[48] Novak B, Tyson JJ. Numerical analysis of a comprehensive model of M-phase control in Xenopus oocyte extracts and intact embryos. J Cell Sci. 1993;106(4):1153–1168. 8126097 10.1242/jcs.106.4.1153

[49] Cross FR, Archambault V, Miller M, Klovstad M. Testing a mathematical model of the yeast cell cycle. Mol Biol Cell. 2002;13(1):52–70. 10.1091/mbc.01-05-0265 11809822 PMC65072

[50] Sha W, Moore J, Chen K, Lassaletta AD, Yi C-S, Tyson JJ, et al Hysteresis drives cell-cycle transitions in Xenopus laevis egg extracts. Proc Natl Acad Sci U S A. 2003;100(3):975–980. 10.1073/pnas.0235349100 12509509 PMC298711

[51] Pomerening JR, Sontag ED, Ferrell JE. Building a cell cycle oscillator: hysteresis and bistability in the activation of Cdc2. Nat Cell Biol. 2003;5(4):346–351. 10.1038/ncb954 12629549

[52] Sabouri-Ghomi M, Ciliberto A, Kar S, Novak B, Tyson JJ. Antagonism and bistability in protein interaction networks. J Theor Biol. 2008;250(1):209–218. 10.1016/j.jtbi.2007.09.001 17950756

[53] Gomez-Uribe C, Verghese GC, Mirny LA. Operating regimes of signaling cycles: statics, dynamics, and noise filtering. PLoS Comput Biol. 2007;3(12). 10.1371/journal.pcbi.0030246 PMC2230677 18159939

[54] Novák B, Tyson JJ. Design principles of biochemical oscillators. Nat Rev Mol Cell Biol. 2008;9(12):981 10.1038/nrm2530 18971947 PMC2796343

[55] Cao Y, Lopatkin A, You L. Elements of biological oscillations in time and space. Nat Struct Mol Biol. 2016;23(12):1030–1034. 10.1038/nsmb.3320 27922613

[56] Batchelor E, Loewer A, Lahav G. The ups and downs of p53: understanding protein dynamics in single cells. Nat Rev Cancer. 2009;9(5):371–377. 10.1038/nrc2604 19360021 PMC2892289

[57] Goldbeter A, Gérard C, Gonze D, Leloup J-C, Dupont G. Systems biology of cellular rhythms. FEBS Lett. 2012;586(18):2955–2965. 10.1016/j.febslet.2012.07.041 22841722

[58] Forger DB. Biological clocks, rhythms, and oscillations: the theory of biological timekeeping. MIT Press; 2017. 31369219

[59] Elowitz MB, Leibler S. A synthetic oscillatory network of transcriptional regulators. Nature. 2000;403(6767):335 10.1038/35002125 10659856

[60] Kim JK, Chen Y, Hirning AJ, Alnahhas RN, Josić K, Bennett MR. Long-range tedatmporal coordination of gene expression in synthetic microbial consortia. Nat Chem Biol. 2019:1–8. 10.1038/s41589-018-0202-5 31611703 PMC6858561

[61] Stricker J, Cookson S, Bennett MR, Mather WH, Tsimring LS, Hasty J. A fast, robust and tunable synthetic gene oscillator. Nature. 2008;456(7221):516 10.1038/nature07389 18971928 PMC6791529

[62] Tigges Marquez-Lago TT, Stelling J, Fussenegger M. A tunable synthetic mammalian oscillator. Nature. 2009;457(7227):309 10.1038/nature07616 19148099

[63] Chen Y, Kim JK, Hirning AJ, Josić K, Bennett MR. Emergent genetic oscillations in a synthetic microbial consortium. Science. 2015;349(6251):986–989. 10.1126/science.aaa3794 26315440 PMC4597888

[64] Ronen M, Rosenberg R, Shraiman BI, Alon U. Assigning numbers to the arrows: parameterizing a gene regulation network by using accurate expression kinetics. Proc Natl Acad Sci U S A. 2002;99(16):10555–10560. 10.1073/pnas.152046799 12145321 PMC124972

[65] Del Vecchio D, Abdallah H, Qian Y, Collins JJ. A blueprint for a synthetic genetic feedback controller to reprogram cell fate. Cell Syst. 2017;4(1):109–120.e11. 10.1016/j.cels.2016.12.001 28065574 PMC5326680

[66] Alon U. An introduction to systems biology: design principles of biological circuits. CRC Press; 2019.

[67] Gonze D, Abou-Jaoudé W. The Goodwin model: behind the Hill function. PLoS ONE. 2013;8(8). 10.1371/journal.pone.0069573 PMC3731313 23936338

[68] Segel LA. Mathematical models in molecular cellular biology. CUP Archive; 1980.

[69] Buchler NE, Cross FR. Protein sequestration generates a flexible ultrasensitive response in a genetic network. Mol Syst Biol. 2009;5(1). 10.1038/msb.2009.30 PMC2694680 19455136

[70] Kim JK, Forger DB. A mechanism for robust circadian timekeeping via stoichiometric balance. Mol Syst Biol. 2012;8(1). 10.1038/msb.2012.62 PMC3542529 23212247

[71] Kim JK. Protein sequestration versus Hill-type repression in circadian clock models. IET Syst Biol. 2016;10(4):125–135. 10.1049/iet-syb.2015.0090 27444022 PMC8687308

[72] Goodwin BC. Oscillatory behavior in enzymatic control processes. Adv Enzym Regul. 1965;3:425–437. 10.1016/0065-2571(65)90067-1 5861813

[73] Griffith J. Mathematics of cellular control processes I. Negative feedback to one gene. J Theor Biol. 1968;20(2):202–208. 10.1016/0022-5193(68)90189-6 5727239

[74] Gonze D, Ruoff P. The Goodwin oscillator and its legacy. Acta Biotheor. 2020:1–18. 10.1007/s10441-020-09379-8 32212037

[75] Kim JK, Kilpatrick ZP, Bennett MR, Josić K. Molecular mechanisms that regulate the coupled period of the mammalian circadian clock. Biophys J. 2014;106(9):2071–2081. 10.1016/j.bpj.2014.02.039 24806939 PMC4017850

[76] D’Alessandro M, Beesley S, Kim JK, Jones Z, Chen R, Wi J, et al Stability of wake-sleep cycles requires robust degradation of the PERIOD protein. Curr Biol. 2017;27(22):3454–3467.e8. 10.1016/j.cub.2017.10.014 29103939 PMC5698108

[77] Lee Y, Chen R. Lee H-M, Lee C. Stoichiometric relationship among clock proteins determines robustness of circadian rhythms. J Biol Chem. 2011;286(9):7033–7042. 10.1074/jbc.M110.207217 21199878 PMC3044960

[78] Ye R, Selby CP, Ozturk N, Annayev Y, Sancar A. Biochemical analysis of the canonical model for the mammalian circadian clock. J Biol Chem. 2011;286(29):25891–25902. 10.1074/jbc.M111.254680 21613214 PMC3138243

[79] Partch CL, Green CB, Takahashi JS. Molecular architecture of the mammalian circadian clock. Trends Cell Biol. 2014;24(2):90–99. 10.1016/j.tcb.2013.07.002 23916625 PMC3946763

[80] Fribourgh JL, Srivastava A, Sandate CR, Michael AK, Hsu PL, Rakers C, et al Dynamics at the serine loop underlie differential affinity of cryptochromes for CLOCK: BMAL1 to control circadian timing. Elife. 2020;9:e55275 10.7554/eLife.55275 32101164 PMC7064333

[81] Lee C, Etchegaray J-P, Cagampang FR, Loudon AS, Reppert SM. Posttranslational mechanisms regulate the mammalian circadian clock. Cell. 2001;107(7):855–867. 10.1016/s0092-8674(01)00610-9 11779462

[82] Narumi R, Shimizu Y, Ukai-Tadenuma M, Ode KL, Kanda GN, Shinohara Y, et al Mass spectrometry-based absolute quantification reveals rhythmic variation of mouse circadian clock proteins. Proc Natl Acad Sci U S A. 2016;113(24):E3461–E3467. 10.1073/pnas.1603799113 27247408 PMC4914154

[83] D’Alessandro M, Beesley S, Kim JK, Chen R, Abich E, Cheng W, et al A tunable artificial circadian clock in clock-defective mice. Nat Commun. 2015;6:8587 10.1038/ncomms9587 26617050 PMC4674671

[84] Rao CV, Arkin AP. Stochastic chemical kinetics and the quasi-steady-state assumption: application to the Gillespie algorithm. J Chem Phys. 2003;118(11):4999–5010.

[85] Cao Y, Gillespie DT, Petzold LR. The slow-scale stochastic simulation algorithm. J Chem Phys. 2005;122(1):014116. 10.1063/1.1824902 15638651

[86] Goutsias J. Quasiequilibrium approximation of fast reaction kinetics in stochastic biochemical systems. J Chem Phys. 2005;122(18):184102 10.1063/1.1889434 15918689

[87] Schnoerr D, Sanguinetti G, Grima R. Approximation and inference methods for stochastic biochemical kinetics—a tutorial review. J Phys A Math Theor. 2017;50(9):093001.

[88] Anderson DF, Craciun G, Kurtz TG. Product-form stationary distributions for deficiency zero chemical reaction networks. Bull Math Biol. 2010;72(8):1947–1970. 10.1007/s11538-010-9517-4 20306147

[89] Sontag ED, Singh A. Exact moment dynamics for feedforward nonlinear chemical reaction networks. IEEE Life Sci Lett. 2015;1(2):26–29.

[90] Kim JK, Sontag ED. Reduction of multiscale stochastic biochemical reaction networks using exact moment derivation. PLoS Comput Biol. 2017;13(6):e1005571 10.1371/journal.pcbi.1005571 28582397 PMC5481150

[91] Mélykúti B, Hespanha JP, Khammash M. Equilibrium distributions of simple biochemical reaction systems for time-scale separation in stochastic reaction networks. J R Soc Interface. 2014;11(97):20140054 10.1098/rsif.2014.0054 24920118 PMC4208355

[92] Sanft KR, Gillespie DT, Petzold LR. Legitimacy of the stochastic Michaelis–Menten approximation. IET Syst Biol. 2011;5(1):58–69. 10.1049/iet-syb.2009.0057 21261403

[93] Kim JK, Jackson TL. Mechanisms that enhance sustainability of p53 pulses. PLoS ONE. 2013;8(6). 10.1371/journal.pone.0065242 PMC3670918 23755198

[94] Kim H, Gelenbe E. Stochastic gene expression modeling with hill function for switch-like gene responses. IEEE/ACM Trans Comput Biol Bioinform. 2011;9(4):973–979. 10.1109/TCBB.2011.153 22144531

[95] Komorowski M, Miękisz J, Kierzek AM. Translational repression contributes greater noise to gene expression than transcriptional repression. Biophys J. 2009;96(2):372–384. 10.1016/j.bpj.2008.09.052 19167290 PMC2716471

[96] Agarwal A, Adams R, Castellani GC, Shouval HZ. On the precision of quasi steady state assumptions in stochastic dynamics. J Chem Phys. 2012;137(4):044105 10.1063/1.4731754 22852595 PMC3416873

[97] Thomas P, Straube AV, Grima R. The slow-scale linear noise approximation: an accurate, reduced stochastic description of biochemical networks under timescale separation conditions. BMC Syst Biol. 2012;6(1):39. 22583770 10.1186/1752-0509-6-39 PMC3532178

[98] Kim JK, Josić K, Bennett MR. The validity of quasi-steady-state approximations in discrete stochastic simulations. Biophys J. 2014;107(3):783–793. 10.1016/j.bpj.2014.06.012 25099817 PMC4129492

[99] Kim JK, Josić K, Bennett MR. The relationship between stochastic and deterministic quasi-steady state approximations. BMC Syst Biol. 2015;9(1):87. 26597159 10.1186/s12918-015-0218-3 PMC4657384

[100] MacNamara S, Bersani AM, Burrage K, Sidje RB. Stochastic chemical kinetics and the total quasi-steady-state assumption: application to the stochastic simulation algorithm and chemical master equation. J Chem Phys. 2008;129(9):09B605. 10.1063/1.2971036 19044893

[101] Barik D, Paul MR, Baumann WT, Cao Y, Tyson JJ. Stochastic simulation of enzyme-catalyzed reactions with disparate timescales. Biophys J. 2008;95(8):3563–3574. 10.1529/biophysj.108.129155 18621809 PMC2553150

[102] Jithinraj P, Roy U, Gopalakrishnan M. Zero-order ultrasensitivity: a study of criticality and fluctuations under the total quasi-steady state approximation in the linear noise regime. J Theor Biol. 2014;344:1–11. 10.1016/j.jtbi.2013.11.014 24309434

[103] Kim JK, Rempala GA, Kang H-W. Reduction for stochastic biochemical reaction networks with multiscale conservations. Multiscale Model Simul. 2017;15(4):1376–1403.

[104] Herath N, Del Vecchio D. Reduced linear noise approximation for biochemical reaction networks with time-scale separation: the stochastic tQSSA+. J Chem Phys. 2018;148(9):094108.

[105] Gómez-Uribe CA, Verghese GC, Tzafriri AR. Enhanced identification and exploitation of time scales for model reduction in stochastic chemical kinetics. J Chem Phys. 2008;129(24):244112 10.1063/1.3050350 19123500 PMC2671675

[106] Parsons TL, Rogers T. Dimension reduction for stochastic dynamical systems forced onto a manifold by large drift: a constructive approach with examples from theoretical biology. J Phys A Math Theor. 2017;50(41):415601.

[107] Gillespie DT. Exact stochastic simulation of coupled chemical reactions. J Phys Chem. 1977;81(25):2340–2361.

[108] Kruskal M, editor. Asymptotology In: Lectures presented at the Trieste Seminar on Plasma Physics. 1965.

[109] Farrow L, Edelson D. The steady-state approximation: fact or fiction? Int J Chem Kinet. 1974;6(6):787–800.

[110] Flach EH, Schnell S. Use and abuse of the quasi-steady-state approximation. Syst Biol (Stevenage). 2006;153(4):187–191. 16986620 10.1049/ip-syb:20050104 PMC2265107

[111] Millat T, Bullinger E, Rohwer J, Wolkenhauer O. Approximations and their consequences for dynamic modelling of signal transduction pathways. Math Biosci. 2007;207(1):40–57. 10.1016/j.mbs.2006.08.012 17070871

[112] Hunding A, Kaern M. The effect of slow allosteric transitions in a simple biochemical oscillator model. J Theor Biol. 1998;191(3):309–322. 10.1006/jtbi.1999.0912 10339398

[113] Kumar A, Josić K. Reduced models of networks of coupled enzymatic reactions. J Theor Biol. 2011;278(1):87–106. 10.1016/j.jtbi.2011.02.025 21377474

[114] Zhou M, Kim JK, Eng GWL, Forger DB, Virshup DM. A Period2 phosphoswitch regulates and temperature compensates circadian period. Mol Cell. 2015;60(1):77–88. 10.1016/j.molcel.2015.08.022 26431025

[115] Narasimamurthy R, Hunt SR, Lu Y, Fustin J-M, Okamura H, Partch CL, et al CK1δ/ε protein kinase primes the PER2 circadian phosphoswitch. Proc Natl Acad Sci U S A. 2018;115(23):5986–5991. 10.1073/pnas.1721076115 29784789 PMC6003379

[116] Gotoh T, Kim JK, Liu J, Vila-Caballer M, Stauffer PE, Tyson JJ, et al Model-driven experimental approach reveals the complex regulatory distribution of p53 by the circadian factor Period 2. Proc Natl Acad Sci U S A. 2016;113(47):13516–13521. 10.1073/pnas.1607984113 27834218 PMC5127372

[117] Brenan KE, Campbell SL, Petzold LR. Numerical solution of initial-value problems in differential-algebraic equations. SIAM; 1996.

